# Hypothalamic effects of progesterone on regulation of the pulsatile and surge release of luteinising hormone in female rats

**DOI:** 10.1038/s41598-017-08805-1

**Published:** 2017-08-14

**Authors:** Wen He, Xiaofeng Li, Daniel Adekunbi, Yali Liu, Hui Long, Li Wang, Qifeng Lyu, Yanping Kuang, Kevin T. O’Byrne

**Affiliations:** 1 Department of Assisted Reproduction, Shanghai Ninth People’s Hospital, Shanghai Jiaotong University School of Medicine, Shanghai, People’s Republic of China; 2 Division of Women’s Health, Faculty of Life Sciences and Medicine, King’s College London, Guy’s Campus, London, UK

## Abstract

Progesterone can block the oestradiol-induced GnRH/LH surge and inhibit LH pulse frequency. Recent studies reported that progesterone prevented premature LH surges during ovarian hyperstimulation in women. As the most potent stimulator of GnRH/LH release, kisspeptin is believed to mediate the positive and negative feedback effects of oestradiol in the hypothalamic anteroventral periventricular (AVPV) and arcuate (ARC) nuclei, while the region-specific role of progesterone receptors in these nuclei remains unknown. This study examined the hypothesis that progesterone inhibits LH surge and pulsatile secretion via its receptor in the ARC and/or AVPV nuclei. Adult female rats received a single injection of pregnant mare serum gonadotropin followed by progesterone or vehicle. Progesterone administration resulted in a significant prolongation of the oestrous cycle and blockade of LH surge. However, microinjection of the progesterone receptor antagonist, RU486, into the AVPV reversed the prolonged cycle length and rescued the progesterone blockade LH surge, while RU486 into the ARC shortened LH pulse interval in the progesterone treated rats. These results demonstrated that progesterone’s inhibitory effect on the GnRH/LH surge and pulsatile secretion is mediated by its receptor in the kisspeptin enriched hypothalamic AVPV and ARC respectively, which are essential for progesterone regulation of oestrous cyclicity in rats.

## Introduction

The positive and negative feedback effects of oestradiol (E_2_) and progesterone are essential in regulating the cyclical activity of the hypothalamic-pituitary-ovarian axis. Acting as a major inhibitory brake in the luteal phase of the ovarian/menstrual cycle, progesterone inhibits gonadotrophin-releasing hormone (GnRH) and luteinising hormone (LH) secretion^1^. Moreover, when administered before or concurrent with E_2_, progesterone inhibits E_2_ positive feedback and abolishes the preovulatory GnRH and gonadotrophin surge. This blockade of the LH surge, observed in many species, including the rat^2^, ewe^3^, monkey^4^ and women^5–7^, is critical for synchronizing the wave of follicular development in the ovary and maintaining the length of luteal phase^8, 9^. Disturbances in progesterone inhibitory feedback have been implicated in infertility associated with enhanced GnRH/LH secretion^10^. Clinically, premature spontaneous LH surges are a major cause of cycle cancellation in women^11^ and recently, progesterone has been shown to successfully prevent premature LH surges in women undergoing ovarian stimulation, thereby validating its use in *in vitro* fertilization (IVF) regimes^6, 7, 12^. Despite its clinical and physiologic importance, the neural mechanisms underlying the inhibitory actions of progesterone on surge, and indeed pulsatile, release of GnRH/LH remain poorly understood.

Although progesterone regulates GnRH secretion via its hypothalamic receptors, the lack of progesterone receptors (PR) on GnRH neurones^2^ suggests its action may involve interneurons expressing sex steroid receptors. The most probable upstream afferents are kisspeptin expressing neurones, which are highly enriched with both oestradiol receptor (ER) and PR^13^, and project directly to the GnRH neurones^14, 15^. Additionally, knockout of PR in kisspeptin neurones in mice, results in loss of LH surges, irregular oestrous cycles and infertility^16, 17^.

In rodent, there are two major populations of kisspeptin neurones: those in hypothalamic anteroventral periventricular nucleus (AVPV) which are thought to underlie the LH surge by mediating positive feedback effect of E_2_, and the arcuate kisspeptin population which coexpress neurokinin B and dynorphin (KNDy) and mediate steroid negative feedback actions on LH secretion^18–20^. In humans, KNDy neurones of the infundibular nucleus^21^ (homologue to the ARC in other species) relay both steroid negative and positive feedback actions^22^. The KNDy neurones are strongly implicated in GnRH pulse generation. Neurokinin B and dynorphin are thought to stimulate and inhibit KNDy neurone activity respectively, driving episodic release of kisspeptin, which in turn drives pulses of GnRH release^23^. Interestingly, ARC kisspeptin neurones may also participate in the regulation of the LH surge. Ablation of KNDy neurones robustly increased the magnitude of the E_2_-induced LH surge in ovariectomised rats^24^, which may be mediated by their projections to the AVPV kisspeptin neurones^25^ or directly to the GnRH neuronal cell bodies in the medial preoptic area (mPOA)^26^ and/or terminals in the median eminence^25^.

Since PR are expressed in virtually all KNDy neurones, they are thought to be critical in mediating progesterone feedback effects on GnRH and LH secretion. Studies in sheep have shown that dynorphin from KNDy neurones may meditate the action of progesterone on GnRH/LH pulsatile frequency^27^. However, the role of hypothalamic PR in control of the LH surge is controversial. Previous studies have indicated that progesterone might act via its receptors in the mPOA to block the LH surge in rats^28^. In contrasts, the expression of PR on kisspeptin neurones is required for the LH surge and normal oestrous cyclicity in mice^16, 17^. Given the afore-mentioned, the role of AVPV and ARC PR in progesterone negative feedback effect on pulsatile LH secretion as well as the LH surge in the rodent remains unclear.

In the present study, we used bilateral microinjections of the potent PR antagonist, RU486, into the AVPV or ARC nuclei to investigate the inhibitory role of progesterone on the LH surge induced by pregnant mare serum gonadotropin (PMSG), which induces follicle development mimicking clinical ovarian stimulation and LH surge induction. Additionally, intra-nuclear administration of RU486 was used to examine the effects of progesterone on pulsatile release of LH and oestrous cyclicity in female Sprague-Dawley rats.

## Results

### Cannulae placement in the AVPV and ARC

**Figure 1:**
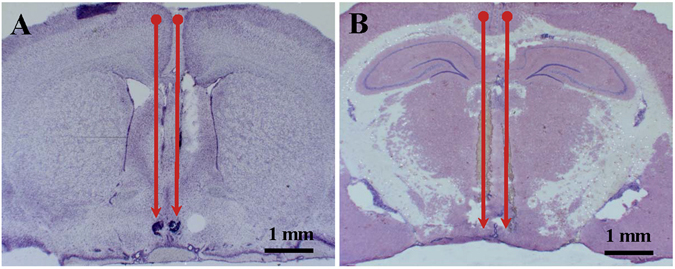
Photomicrograph of the cannulae targeted sites in the hypothalamic anteroventral periventricular (AVPV) and the arcuate (ARC). Photomicrograph of cresyl-violet stained coronal section showing representative examples of bilateral cannula placement in the AVPV (**A**) and ARC (**B**) respectively. Magnification, ×1.25. The arrows indicate the site of cannula placement.

The location of the intra-AVPV and intra-ARC cannulae were confirmed by microscopic histological inspection of cresyl‐violet stained brain sections. Only animals with appropriate bilateral cannulae placement in the AVPV or ARC were included in the analysis (Fig. 1). Of the 16 rats that underwent hypothalamic cannulation, 13 and 12 were confirmed with correct bilateral cannulae in the AVPV or ARC, respectively. The remaining rats were excluded from the analysis due to inaccurate probe placement.

### Effects of progesterone on ovarian cyclicity

**Figure 2:**
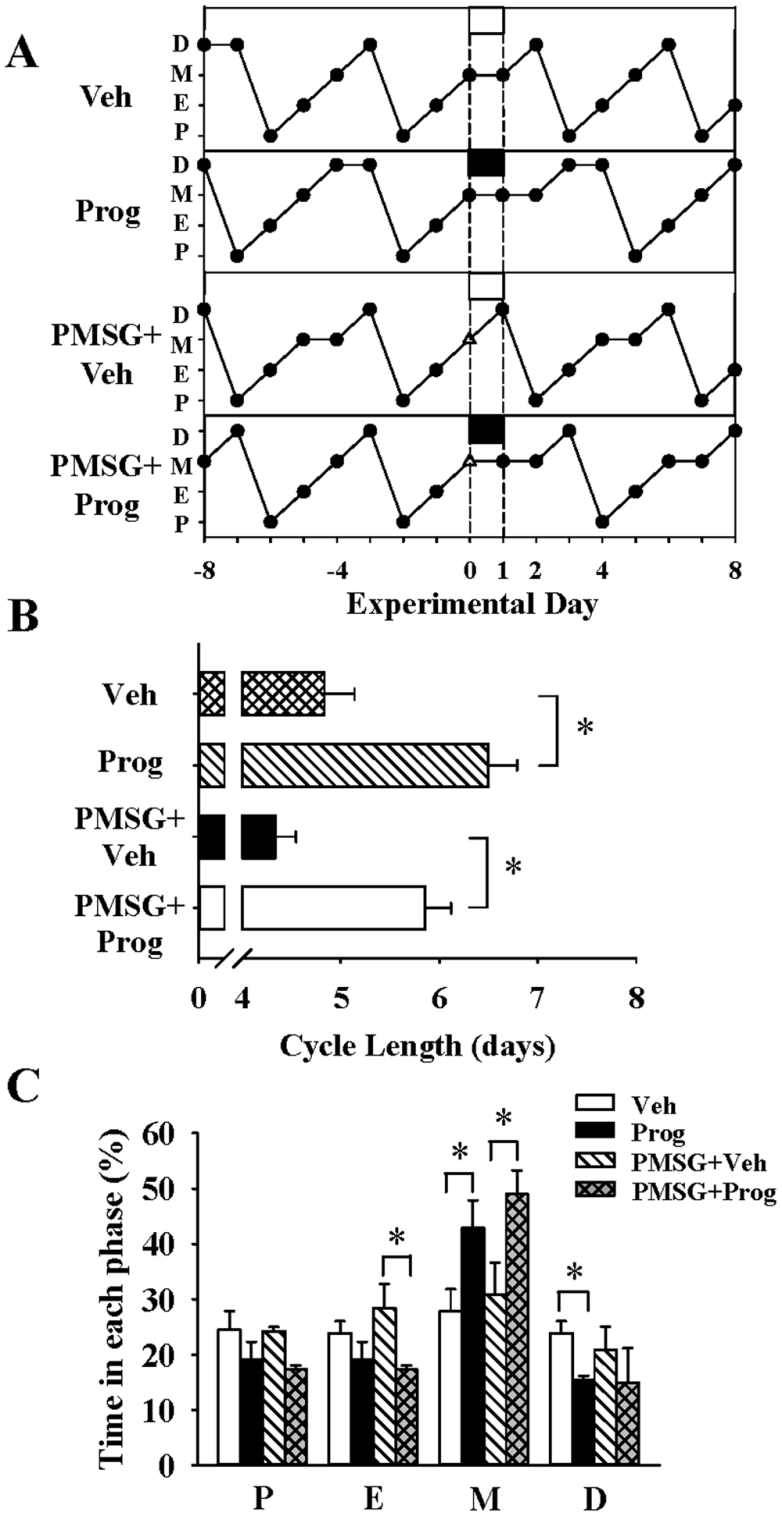
Progesterone disrupted oestrous cyclicity in the female rats. (**A**) Representative profiles depicting oestrous cyclicity in female rats, as measured by vaginal cytology, before (days −8 to −1, baseline), during (day 0 to 1, treatment) and after (days 2 to 8, after-treatment) the two day period of treatment from metestrus with vehicle (oil; twice daily) only (Veh, top panel), progesterone (twice daily) only (Prog, second panel), single injection of PMSG (day 0 only) plus vehicle (twice daily) (PMSG + Veh) or single injection of PMSG (day 0 only) plus progesterone (twice daily) (PMSG + Prog, bottom panel) in normal cycling rats. P, proestrus; E, oestrus; M, metestrus; D, diestrus. (**B**) Average oestrous cycle length of the treatment cycle *per se*. (**C**) Average time spent in each stage of the cycle of the treatment cycle *per se*. Data were analysed by two-way ANOVA. *P < 0.05, Veh vs Prog; n = 5–7 per group.

To test whether exposure to progesterone influences ovarian cyclicity, progesterone was administered on metestrus to normal cycling rats, as well as to pregnant mare serum gonadotropin (PMSG) primed rats to mimic an ovarian stimulation background as described in the method below. In the normal cycling rats, vaginal cytology reveals that the progesterone treatment prolongs cycle length (Progesterone vs Vehicle: 6.71 ± 0.67 vs 4.68 ± 0.35 d; n = 7 and 5, respectively; P < 0.05) (Fig. 2B), with a significant increase in time spent in metestrus but decrease in the diestrus phase (P < 0.05) (Fig. 2C). Representative examples of typical oestrous cyclicity in vehicle and progesterone treated animals and shown in Fig. 2A. Similarly, in PMSG-primed animals, progesterone administration lengthened the oestrous cycle (PMSG + Progesterone vs PMSG + Vehicle: 5.57 ± 0.81 vs 4.50 ± 0.71 d; n = 7 and 5, respectively; P < 0.05) (Fig. 2B), especially the metestrus phase (Fig. 2C). Of note, all vehicle injected PMSG-primed animals were proestrus on day 2 (day 0, PMSG treatment), whereas proestrus was delayed in the progesterone treated rats, and representative examples are illustrated in Fig. 2A.

### Effects of progesterone on the LH surge in PMSG-primed rat

**Figure 3:**
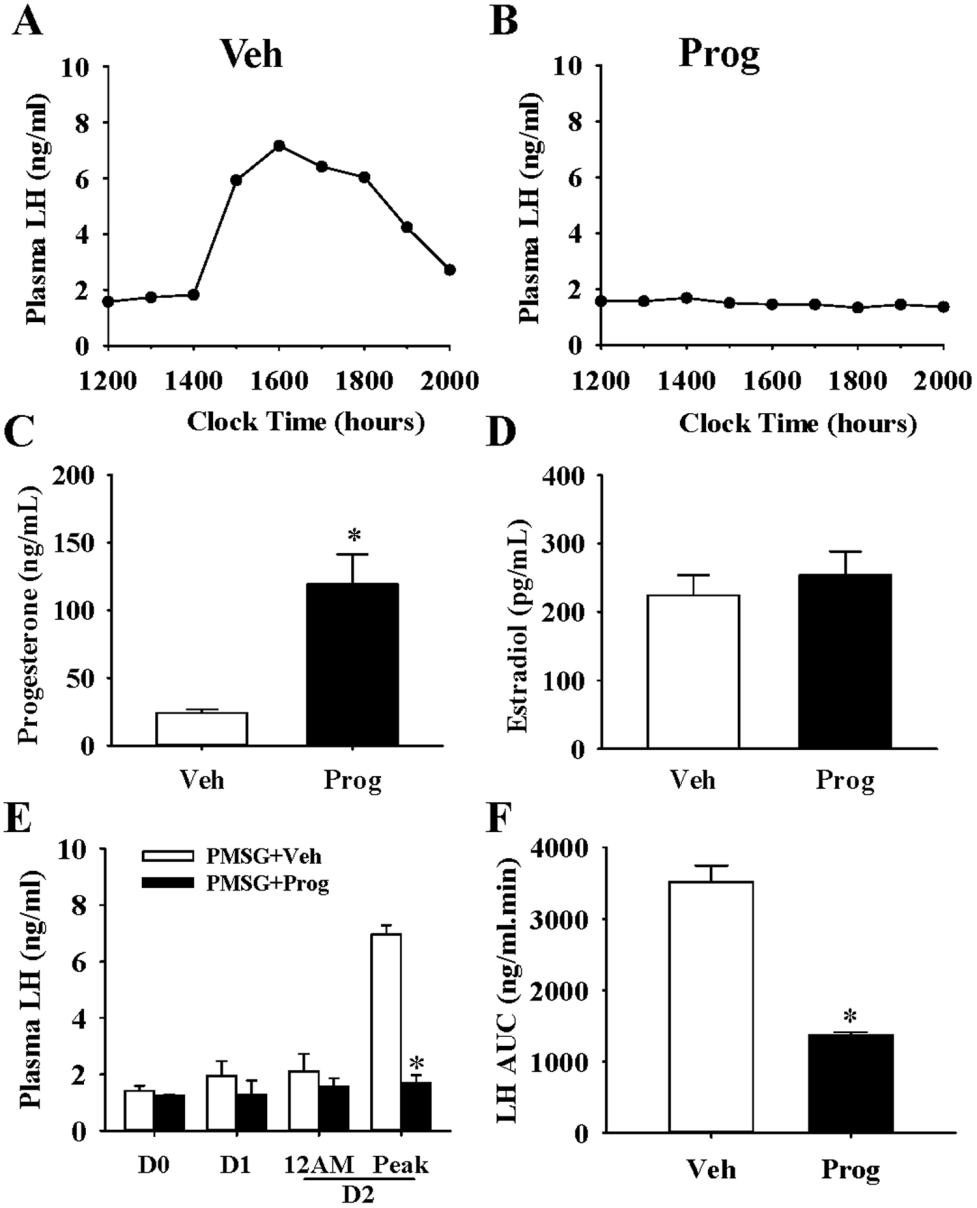
Progesterone disrupted the LH surge in PMSG-primed female rats. Representative examples illustrating levels of LH in oil vehicle (Veh, **A**) or progesterone (Prog, **B**) treated animals at the expected time of the PMSG-induced LH surge on the afternoon of day 2. (**C**) Mean serum progesterone measured at 18:00 h on day 0 (day 0, PMSG treatment) after injection with vehicle or progesterone in PMSG-primed female rats. (**D**) Mean serum oestradiol level measured at 18:00 h on day 1 (day 0, PMSG treatment) after injection with vehicle or progesterone in PMSG-primed female rats. (**E**) Significant difference in peak LH levels at the time of the expected LH surge between oil controls and progesterone treated PMSG-primed animals. Baseline LH level at noon (12AM) on day 2 showed no difference. (**F**) Area under curve (AUC) analysis of LH levels at the time of the expected LH surge (12:00–20:00 h, day 2) in PMSG-primed female rats treated with oil vehicle or progesterone revealed a significant deference; n = 6 rats/group. Values were analysed by two-way ANOVA with group (Veh vs Prog). Results are presented as means ± SEM. *P < 0.05, Veh vs Prog; n = 5–7 per group.

As expected from the proestrus smear, all vehicle injected PMSG-primed animals displayed a robust LH surge on the afternoon/evening of day 2 (day 0, PMSG treatment) (Fig. 3A,E and F). In contrast, none of the PMSG-primed females administered progesterone displayed an LH surge (Fig. 3B,E and F). Area under curve (AUC) analysis of the LH profile (12:00–20:00 h) on day 2 is shown in Fig. 3F.

Circulating progesterone levels were significantly elevated at 18:00 h on experimental day 0 post injection (124.62 ± 43.62 vs 22.79 ± 5.64 ng/ml vehicle control; P < 0.05) (Fig. 3C). Circulating levels of E_2_ measured on the afternoon of day 1, before the expected LH surge, as a measure of follicle development, did not differ between PMSG-primed female treated with vehicle or progesterone (224.1 ± 66.1 vs 254.2 ± 76.3 pg/ml, respectively; P > 0.05) (Fig. 3D).

### Effects of progesterone receptor antagonism in the AVPV or ARC nuclei on LH surges and oestrous cyclicity in PMSG-treated rats

**Figure 4:**
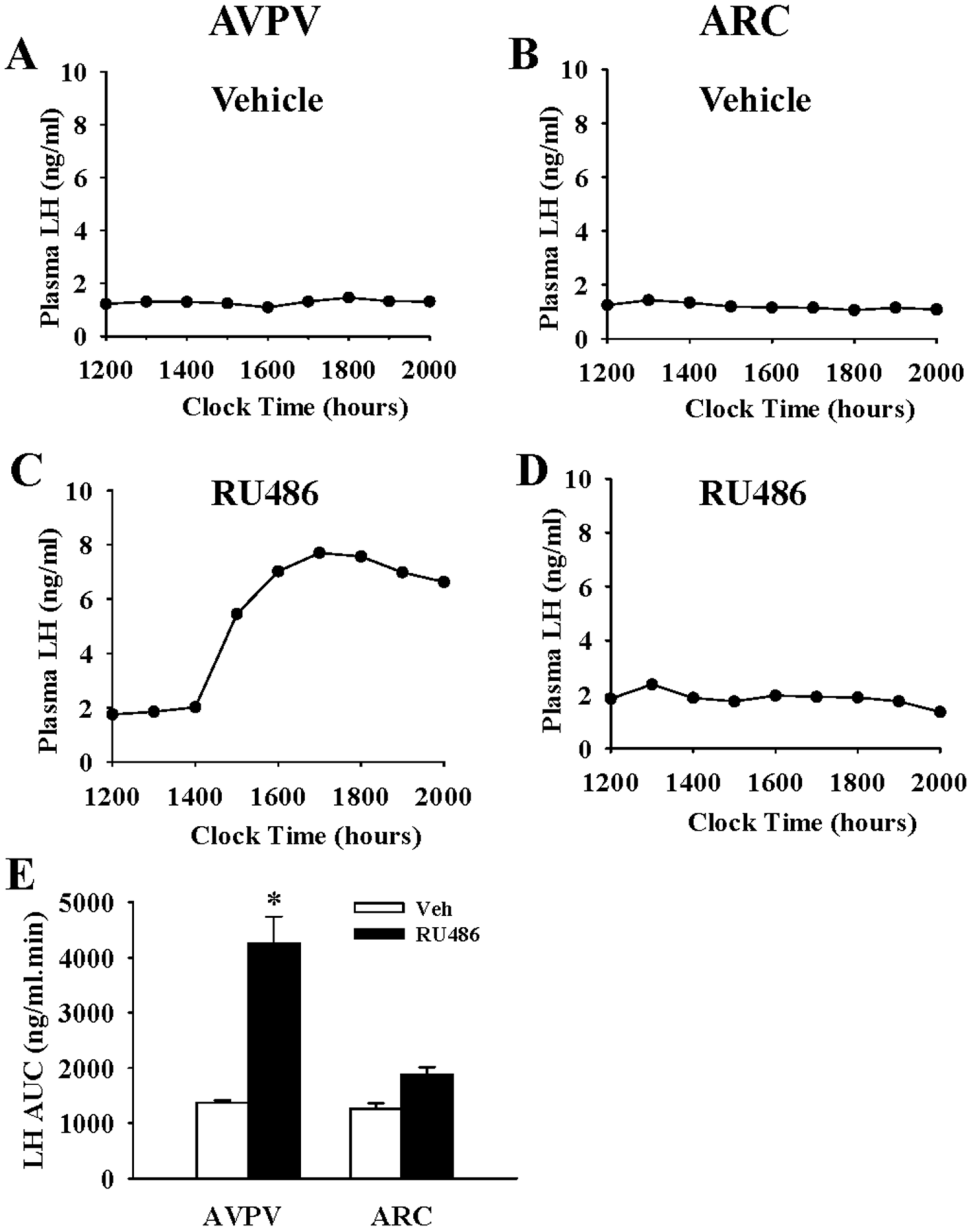
Micro-infusion of RU486 into the AVPV reversed the inhibitory effects of progesterone on the LH surge in PMSG-primed female rats. Representative LH surge on the afternoon of day 2 (day 0, PMSG treatment) in response to intra-AVPV RU486 infusion in a progesterone treated animal (**C**). Intra-AVPV infusion of vehicle (artificial cerebrospinal fluid/DMSO/ethylene glycol) failed to reverse the inhibitory effect of progesterone on the LH surge (**A**). Representative examples showing the absence of LH surges on the afternoon of day 2 following intra-ARC vehicle control (**B**) or RU486 (**D**) after progesterone priming. (**E**) AUC analysis of LH level on the afternoon of day 2 after intra-AVPV or intra-ARC administration with vehicle (Veh) or RU486 in PMSG-treated animals. Results are presented as means ± SEM. *P < 0.05, Veh vs RU486; n = 6–7 per group.

To determine whether progesterone signalling within the AVPV or ARC may play a role in its inhibitory feedback effects on the LH surge and oestrous cyclicity, the progesterone receptor antagonist, RU486, was micro-infused into these brain areas in progesterone treated PMSG-primed animals described above. Administration of RU486 into the AVPV rescued the PMSG stimulated LH surge on day 2 (day 0, PMSG treatment) in 5 out of 7 animals (Fig. 4C), while no LH surge was evident in controls (n = 6) (Fig. 4A and E). In contrast, intra-ARC administration of RU486 failed to rescue the PMSG stimulus LH surge on day 2 (P > 0.05; n = 6 per group) (Fig. 4B,D and E). A summary of these data with AUC analysis of LH levels is provided in Fig. 4E.

**Figure 5:**
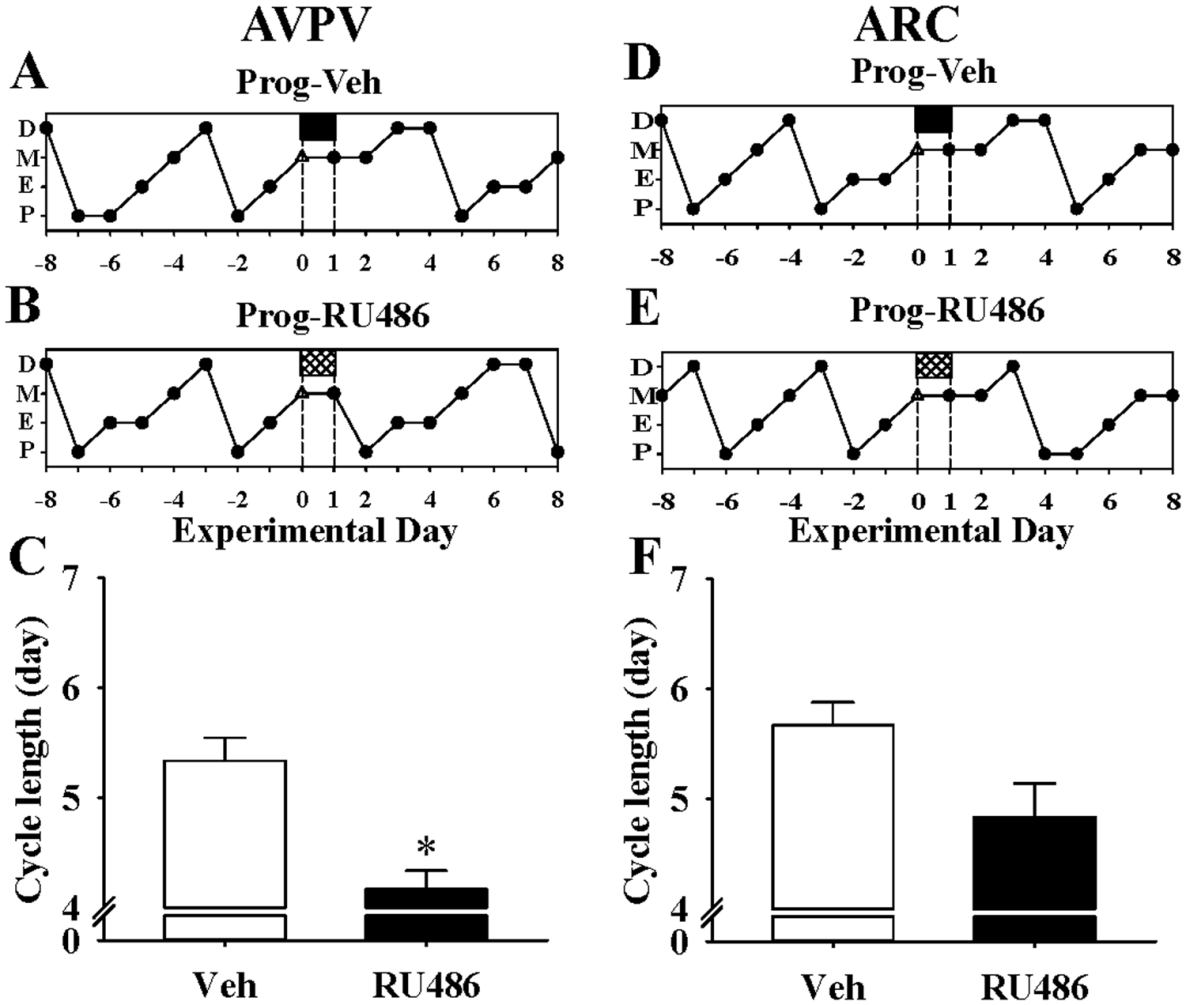
Effects of RU486 micro-infusion into the AVPV or ARC on progesterone induced prolongation of oestrous cycle length in PMSG-primed female rats. RU486 or vehicle (Veh) was injected via the AVPV or ARC nuclear cannulae an hour before each progesterone injection in PMSG-primed rats. Representative examples illustrating the effects of intra-AVPV micro-infusion of vehicle (Prog-Veh, **A**) or RU486 (Prog-RU486, **B**) on oestrous cycle length. (**C**) Intra-AVPV administration of RU486 before progesterone significantly shortened oestrous cycle length compared with vehicle controls. Representative examples illustrating the effects of intra-ARC micro-infusion of vehicle (Prog-Veh, **D**) or RU486 (Pro-RU486, **E**) on oestrous cycle length. (**F**) Oestrous cycle length was not significantly altered by intra-ARC RU486 infusion compared with vehicle controls. Results are presented as means ± SEM. *P < 0.05, Veh vs Prog, n = 6–7 per group.

To verify the effects of progesterone on oestrous cyclicity, daily vaginal cytology was examined. Compared with vehicle infusion, bilateral intra-AVPV administration of RU486 significantly decreased cycle length in the presence of progesterone (P < 0.05; n = 6–7 per group) (Fig. 5A,B and C). However, intra-ARC administration of RU486 showed only a tendency to reduce cycle length compared with vehicle (P > 0.05; n = 6 per group) (Fig. 5D,E and F).

### RU486 restores LH pulse frequency in progesterone treated female rats

**Figure 6:**
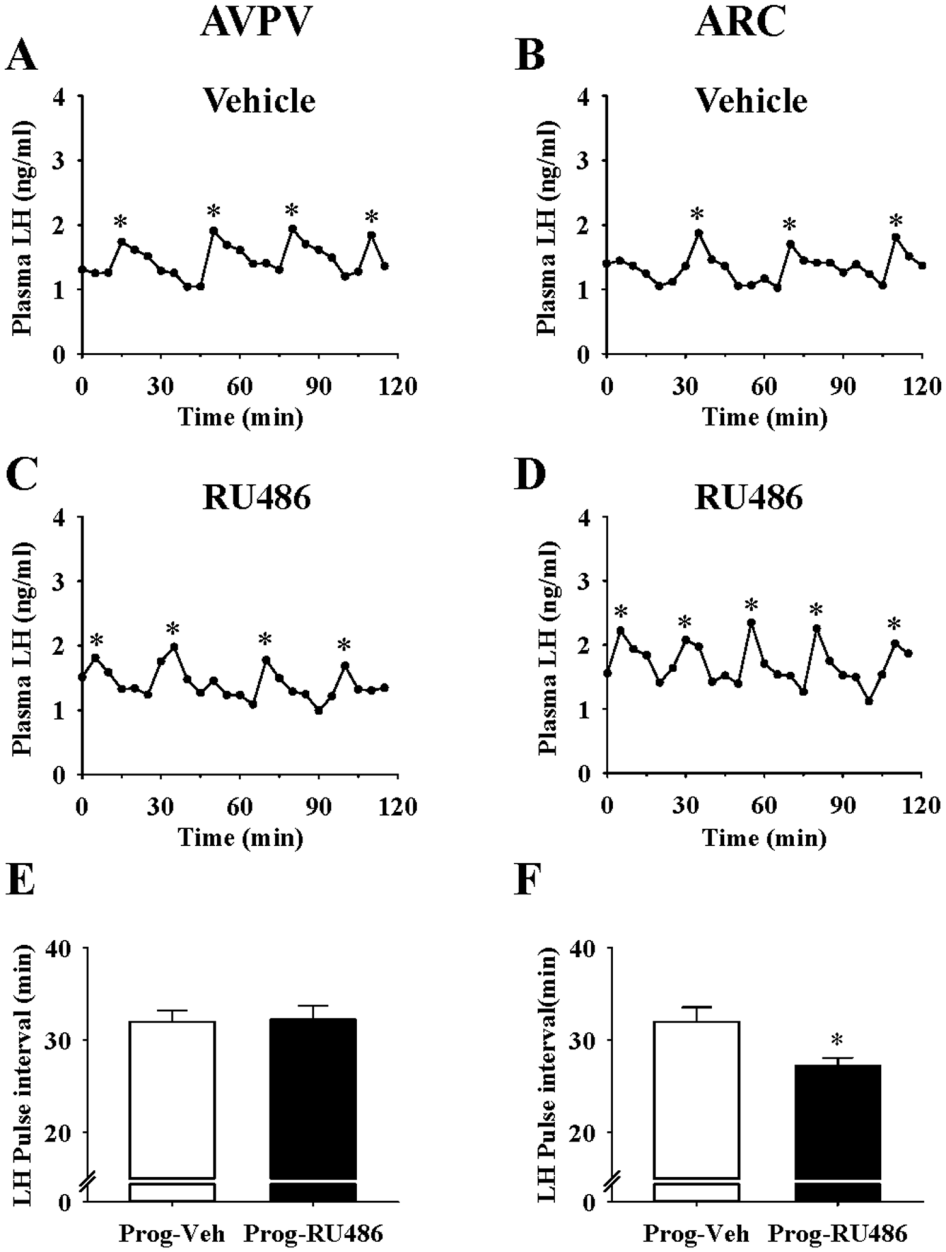
Micro-infusion of RU486 into the ARC, but not the AVPV, reversed the inhibitory effects of intraperitoneal injection of progesterone on LH pulse frequency in female rats. Representative examples illustrating the effects of intra-AVPV administration of vehicle (artificial cerebrospinal fluid/DMSO/ethylene glycol) (**A**) or RU486 (**C**) on LH pulses in progesterone treated animals. (**E**) LH pulse interval was not altered by intra-AVPV administration of RU486 and compared with vehicle. Representative LH profiles illustrating the effects of intra-ARC administration of vehicle (**B**) or RU486 (**D**) in progesterone treated animals. (**F**) Intra-ARC RU486 infusion significantly decreased LH pulse interval compared with vehicle controls. Results are presented as means ± SEM. *P < 0.05, Prog-Veh vs Prog-RU486; n = 6–7 per group. LH pulses are indicated by the asterisk (*).

To investigate the site of the inhibitory action of progesterone on LH pulse frequency, RU486 was bilaterally infused into the AVPV or ARC. Intra-ARC administration of RU486 significantly reduced LH pulse interval compared with controls (P < 0.05; n = 6–7 per group) (Fig. 6B,D and F). In contrast, intra-AVPV administration of RU486 had no effect on LH pulse interval in the presence of progesterone (P > 0.05; n = 6 per group) (Fig. 6A,C and E). LH pulse amplitude was not affected in any treatment group (data not shown).

## Discussion

The present study provides direct evidence of a role for PR in the hypothalamic AVPV and ARC nuclei in mediating the inhibitory effects of progesterone on surge and pulsatile release of LH, and oestrous cyclicity in female rats. Intra-AVPV administration of the progesterone antagonist, RU486, attenuated the inhibitory effect of progesterone on LH surges in PMSG-primed female rats, while antagonism of PR in the ARC restored LH pulse frequency in progesterone-treated animals.

Previous studies have shown that an increase in circulatory level of progesterone between oestrous to metestrus is related to prolonged oestrous cycle length in rats^29, 30^. Nequin L. G. *et al*.^30^ reported that higher endogenous serum progesterone level (around 88.5 ng/ml) after ovulation resulted in longer cycle length in rats. In accordance with these data, we confirm that exogenous administration of progesterone (serum level 124.62 ± 43.62 ng) on metestrus prolonged oestrous cycle lengths, particularly the time spent in metestrus stage. PMSG has previously been used to stimulate and synchronize follicular growth and ovulation, mimicking ovarian stimulation in women^31, 32^. In the present study, progesterone extended oestrous cycle length, regardless of the prevailing oestrogenic milieu stimulated by PMSG. Everett *et al*.^29^ reported cycle prolongation by progesterone was accompanied by delayed ovulation, suggesting an antagonistic role of progesterone on oestrogenic stimulation of ovulation. Steroid feedback on LH surge^20^ and pulse^19^ generation are mediated by kisspeptin signalling in the AVPV and ARC respectively. Kisspeptin neurone specific ER knockout mice arrest between the oestrous and diestrus phases and are anovulatory^33^. Similarly, antagonism of GnRH signalling inhibits spontaneous ovulation and oestrous cyclicity^34^. These data suggest that cycle arrest in metestrus and diestrus is accompanied by interruption of E_2_ feedback via kisspeptin on GnRH/LH secretion which is essential for ovulation^33^. Indeed, moderate knockdown of kisspeptin signalling in the AVPV, results in extended cycle length, and increased the time spent in metestrus and/or oestrus^20^. Of note, the present study shows that progesterone antagonism within the AVPV reversed the prolonged oestrous cycle length induced by progesterone. Additionally, PR knockout in kisspeptin neurones in adult female mice shows abnormal cycle with persistent diestrus and consequently anovulatory^16^. It therefore appears that progesterone may interrupt oestrogen-kisspeptin-GnRH signalling in the AVPV, thus affecting ovulation and reproductive cycles.

Progesterone can either facilitate or inhibit E_2_-induced LH surges, depending on their temporal relationship. When administered after E_2_ priming, progesterone augments and synchronizes the preovulatory LH surge^35^, whereas high levels of progesterone, as during luteal phase of oestrous/menstrual cycle, block the stimulatory effects of E_2_ on LH surges in rats^2^, sheep^3, 36^ and primates^4^, including human^5^. In the present study, our data are consistent with these previous findings and demonstrates the inhibitory effect of progesterone on the GnRH/LH surge under a high E_2_ milieu induced by PMSG in female rats. Of note, these results are in line with our observation of prolonged cycle after administration of progesterone to mimic the component of the luteal phase of the human menstrual cycle. Indeed, the ability of progesterone to blockade the preovulatory LH surge is the basis of the progesterone primed ovarian stimulation regime widely adopted for infertility patient in IVF clinic. Either endogenous progesterone in the luteal phase^6, 37^ or exogenous progesterone in follicular phase^7^ is sufficient to block the LH surge without compromising oocyte competence for women during ovarian stimulation.

Previous studies have shown that progesterone inhibits E_2_–induced GnRH release^38^ and decreases GnRH output in response to electrical stimulation of the mPOA^39^. Although early studies pointed to the hypothalamic preoptic area as a possible site for mediating progesterone inhibition of the LH surge^28^, there are no PR expressed in mPOA GnRH neurones^2^. However, kisspeptin neurones are enriched with both PR and ER^40, 41^, and project directly to the mPOA GnRH neurones^14^, suggesting they are the target of progesterone. It is generally accepted that E_2_ acts via ER in the AVPV kisspeptin neurones to activate GnRH neurones resulting in the LH surge^40^. However, there is yet no direct evidence for the specific role of PR in the AVPV kisspeptin neurones in regulating the LH surge. In the present study, we confirm the blockade of LH surges by progesterone, and further provide evidence for the site specific role of PR in the AVPV in mediating the inhibitory effect of progesterone on LH surges, based on the observation that progesterone antagonism within the AVPV rescues LH surges blocked by progesterone. A role of PR in the ARC seems inhibitory because intra-ARC administration of RU486 failed to rescue the inhibitory effect of progesterone on the LH surge. These data are in keeping with the role of AVPV kisspeptin in mediating the E_2_-induced LH surge^20^. Additionally, PR knockout in kisspeptin neurones result in blockade of E_2_-induced LH surges and lose of oestrous cyclicity in mice^16, 17^. It therefore appears that progesterone inhibits the pre-ovulatory LH surge by interrupting activation of the AVPV kisspeptin neurones to elicit the GnRH/LH surge. Paradoxically, the ability of E_2_ to induce the LH surge is dependent on the presence and activation of PR^42^. While E_2_ induces PR expression in the AVPV^43^, progesterone down regulates its own receptors^44, 45^ and may counteract the induction of PR by E_2_. Blockade of the LH surge by progesterone may therefore result from PR in the AVPV, thereby attenuating the ability of E_2_ to induce the LH surge.

The present study indicates that the ARC is a crucial site for progesterone’s inhibitory effect on LH pulse frequency in keeping with the role of this region in LH pulses generation^20^. Intra-ARC injection of RU486 significantly increased LH pulse frequency in progesterone treated rats. This result supports the findings of Goodman *et al*.^46^, where local antagonism of PR in the ARC interfered with the action of progesterone in reducing LH pulse frequency in sheep. As a critical component of the GnRH pulse generator, the ARC KNDy neurones are highly enriched with PR^13^, and have direct projections to GnRH cell bodies^26^ and terminals^25^. It has been reported that dynorphin released by KNDy neurones inhibit pulsatile GnRH secretion and may mediate progesterone negative feedback particularly in ewe^27, 47^, and a similar mechanism may operate in the rodent. Given the inhibitory role of dynorphin on LH secretion, it is suggested that progesterone may be act on ARC KNDy neurones and through dynorphin release inhibits pulsatile GnRH and LH secretion.

In conclusion, these data provide evidence that the inhibitory effect of progesterone on preovulatory surge and pulsatile secretion of LH is mediated by its receptor in the kisspeptin enriched hypothalamic AVPV and ARC respectively, which are essential for progesterone regulation of ovarian cyclicity.

## Methods

### Animals and surgical procedures

Female Sprague-Dawley rats weighing 150–200 g, obtained from Charles-Rivers (Margate, UK), were housed individually under controlled temperature (22 ± 2 °C), light conditions (12:12 h light/dark, with lights on at 07:00 h) and fed with standard laboratory chow and water ad libitum. All surgical procedures were carried out under ketamine (100 mg/kg i.p.; Pharmacia and Upjohn, Crawley, UK) and Rompun (10 mg/kg i.p.; Bayer, Leverkusen, Germany) anaesthesia. All procedures were conducted in accordance with the British Home Office Animals Scientific Procedures Animals Act 1986 (Project Licence 70/6237) and all experimental protocols were approved by the Animal Welfare and Ethical Review Body at King’s College London.

### Jugular vein cannulation

Animals were implanted with two custom-made cardiac catheters via the jugular veins to enable serial blood sampling for profiling of LH levels^48^. The catheters were exteriorised at the back of the head and enclosed within a 30-cm light-weight metal spring tether (Instec Laboratories, Boulder, CO, USA) secured to the slotted screw (Instec Laboratories) affixed to the surface of the skull using dental cement (Dental Filling Ltd., Swindon, UK). The distal end of the tether was attached to a fluid swivel (Instec Laboratories), which allowed the rat freedom to move around the enclosure. After surgery, all rats were housed individually and experiments commenced about 3 days later.

### Bilateral AVPV or ARC cannulae implantation

To assess the effect of the anti-progesterone, RU486, in the hypothalamic AVPV or ARC nuclei, animals were secured in a David Kopf stereotaxic frame and implanted with bilateral guide cannulae (33-gauge; Plastics One, Roanoke, VA, USA) targeted towards the AVPV or ARC for later microinjection of drug. The respective coordinates for the ARC and AVPV were 0.40 mm lateral, 0.00 mm posterior to bregma, 8.60 mm below the surface of the dura, and 0.50 mm lateral, 3.40 mm anterior to bregma, 10.10 mm below the surface of the dura according to the rat brain atlas of Paxinos and Watson^49^. A stainless steel slotted screw (Instec Laboratories) was affixed to the surface of the skull posterior to the guide cannulae and both were secured using dental cement (Dental Filling). The guide cannulae were then fitted with obturators (Plastics One) to maintain patency. After a 7-day recovery period the rats were implanted with cardiac catheters as described above. Experimentation commenced after a further 3-day recovery from surgery.

### Hormone treatment

Treatment with PMSG has been validated for induction of follicle development to mimic ovarian stimulation^50, 51^ and induction of the LH surge^32^, and facilitates study of mechanisms underlying these key reproductive processes. To test the action of progesterone on the LH surge female rats receiving a single injection of PMSG (150 IU/kg, i.p., Sigma-Aldrich, Poole, UK) on the morning of metestrus, which results in an LH surge approximately 55 h later^31, 32^, were administered progesterone (5 mg/kg, i.p., in peanut oil, Sigma-Aldrich) twice daily (at 09:00 and 17:00 h) for two consecutive days starting immediately after the PMSG injection. For controls, PMSG-primed rats were injected with vehicle (0.3 ml, peanut oil, i.p., Sigma-Aldrich).

As an additional control group, to evaluate the effect of progesterone *per se* in normal cycling rats, progesterone or vehicle only was given twice daily for two days starting on metestrus as described above.

### Effects of progesterone on oestrous cyclicity

Vaginal lavage was performed daily between 07:00 and 08:00 h to detect the stages of the oestrous cycle; proestrus, oestrus, metestrus and diestrus. Rats that displayed at least two consecutive 4- to 5-day oestrous cycles, with positive classification for all 4-stages were used. After at least one control oestrous cycle, PMSG-primed (n = 12) and non-PMSG (n = 10) treated control rats were randomly assigned on the morning of metestrus to receive either progesterone (5 mg/kg) or oil vehicle (0.3 ml) injections twice daily for 2 days as described above. Oestrous cyclicity was monitored for a further 8–10 days. The oestrous cycle length was calculated as the number of days between successive occurrences of oestrus. The time spent in each cycle stage was calculated as the proportion of days classified in each cycle stage.

### Effects of progesterone on the LH surge in PMSG-primed rats

On the morning of day 2 (day 0, PMSG treatment) at 11:00 h the PMSG-primed animals were attached via one of the two cardiac catheter to a computer-controlled automated blood sampling system, which allows for the intermittent withdrawal of 25-µl blood samples without disturbing the animals^48^. Once connected, animals were left undisturbed for 1 h before sampling commenced at 12:00 h, when samples were collected hourly for 8 h (till 20:00 h) for LH measurement. After removal of each 25-µl blood sample, an equal volume of heparinized saline (50 U/ml heparin sodium/ml normal saline; CP Pharmaceuticals, Wrexham, UK) was automatically infused into the animal to maintain patency of the catheter and blood volume. Blood samples were frozen at −20 °C for later assay to determine LH concentrations. Blood samples (25-µl) were also collected at 18:00 h on day 0 and day 1 for LH measurement during experiment. Additional blood samples (200 µl) were collected at 18:00 h on days 0 and 1 for progesterone and E_2_ measurement, respectively, thus confirming hormone injection and status of follicular development.

### Effects of progesterone receptor antagonism in the AVPV or ARC nuclei on LH surges and oestrous cyclicity in PMSG treated rats

A separate group of animals implanted with guide cannulae in the AVPV (n = 16) or ARC (n = 16) nuclei and cardiac catheters were monitored for normal oestrous cyclicity and then treated with the identical PMSG and progesterone regime described above. To examine the role of AVPV and ARC nuclear PR in surge release of LH, rats received bilateral intra-AVPV or intra-ARC injections of the anti-progesterone, RU486 (4 ng in 0.8 μl; Sigma‐Aldrich) or vehicle (0.8 μl, 60% artificial cerebrospinal fluid (aCSF), 20% DMSO and 20% ethylene glycol; Sigma‐Aldrich) 1 h before each intraperitoneal injected of progesterone (i.p. at 08:00 and 16:00 h for 2 days starting on metestrus). Internal cannulae (Plastics One) with extension tubing, preloaded with RU486 or vehicle, were inserted into the guide cannulae and extended 1.0 mm beyond the tips to reach the AVPV or ARC nuclei. The distal end of the extension tubing was connected to a 5‐μl syringe (SGE Analytical Science, Milton Keynes, UK). The tubing was held outside the cage to allowing remote infusion without disturbing the animal. The RU486 or vehicle was micro-infused at a rate of 0.1 μl/min for 8 min and the internal cannulae were kept in the guide cannulae for a further 2 min to prevent backflow. This procedure was repeated for each of the 4 injections.

At 11:00 h on day 2 (day 0, PMSG treatment) the animals were attached via a cardiac catheter to the blood sampling system, and left undisturbed for 1 h before sampling commenced at 12:00 h. Samples were collected hourly for 8 h for LH measurement as described above.

Oestrous cyclicity was monitored in this experimental group by examining daily vaginal cytology.

### Effects of microinjection of RU486 into the AVPV or ARC on LH pulse

On completion of the experiments described above, the same animals were used to examine the role of AVPV and ARC nuclear PR in pulsatile release of LH, after a washout period of about two weeks during which recovery of normal oestrous cyclicity was observed. At 08:00 h on morning of metestrus, rats were given bilateral intra-AVPV or intra-ARC injection of RU486 (4 ng in 0.8 μl: Sigma‐Aldrich) or vehicle (0.8 μl, 60% artificial cerebrospinal fluid (aCSF), 20% DMSO and 20% ethylene glycol; Sigma‐Aldrich) as described above. One hour later (09:00 h), rats received an intraperitoneal injection of progesterone (5 mg/kg), and automated blood sampling commenced, as previously described, 3 h later (12:00 h) with collection of 25-μl blood every 5 min for 2 h for LH measurement. All the samples were frozen at −20 °C for later assay.

### Brain collection and histological verification of cannulae position

After experimentation, 0.5 µl India ink was injected through internal cannulae inserted into the guide cannulae for the purpose of site verification. Animal were then killed by decapitation. The brains were removed and snap frozen on dry ice, and then stored at −80 °C followed by sectioning (30-µm) using a cryostat (Bright Instrument Co Ltd., Luton, UK). To evaluate the cannulae position, every fourth section throughout the AVPV or ARC region corresponding to bregma −0.60 to 0.80 mm and 2.80 to 3.60 mm^49^, respectively, was mounted and stained with cresyl violet. Slides were then viewed under a light microscope and images were taken using a digital camera (Zeiss, Oberkochen, Germany). Only data from animals with correct cannulae placement were analysed.

### Measurement of plasma LH, oestradiol and progesterone concentration

A double-antibody radioimmunoassay (RIA) supplied by the National Institute of Diabetes and Digestive and Kidney Diseases (Bethesda, MD, USA) was used to determine LH concentration in the 25-μlwhole blood sample^52^. The reference preparation was rLH-RP-3. The sensitivity of the assay was 0.093 ng/ml. The intra-assay coefficient of variation was 6.5% and the inter-assay coefficient of variation was 7.4%. The E_2_ and progesterone plasma samples (50 μl) were measured by using the EIA kit (Labor Diagnostika Nord, Germany) which use was verified in rat^53^.

### Statistical analysis

The effect of progesterone and RU486 on LH surges was determined by analysing the area under the LH profile curve (AUC) using Sigma Plot 12.0 (Systat Software, Inc., Chicago, IL). Detection of LH pulses was established through the use of the algorithm ULTRA^54^. Two intraassay coefficients of variation of the assay were used as the reference threshold for the pulse detection. The effect of intra-AVPV and intra-ARC RU486 microinjection on parameters of LH secretion was calculated by comparing the mean basal levels of LH, mean LH pulses interval, or mean amplitude of LH pulses, within the experimental period between the two groups. A Student’s *t* test was used to analyse baseline LH, E_2_ and progesterone levels. All other results were analysed by a one-way ANOVA. Data are presented as the mean ± SEM and P < 0.05 was considered statistically significant.
